# Supplementary material for: Novel Substituted Azoloazines with Anticoagulant Activity
Source: Int J Mol Sci. 2023 Oct 25;24(21):15581. doi: 10.3390/ijms242115581 (PMC10648877; doi:10.3390/ijms242115581)
Supplement: Supplementary file 1 [file ijms-24-15581-s001.zip › ijms-2669368-supplementary.pdf]

Supplementary Materials

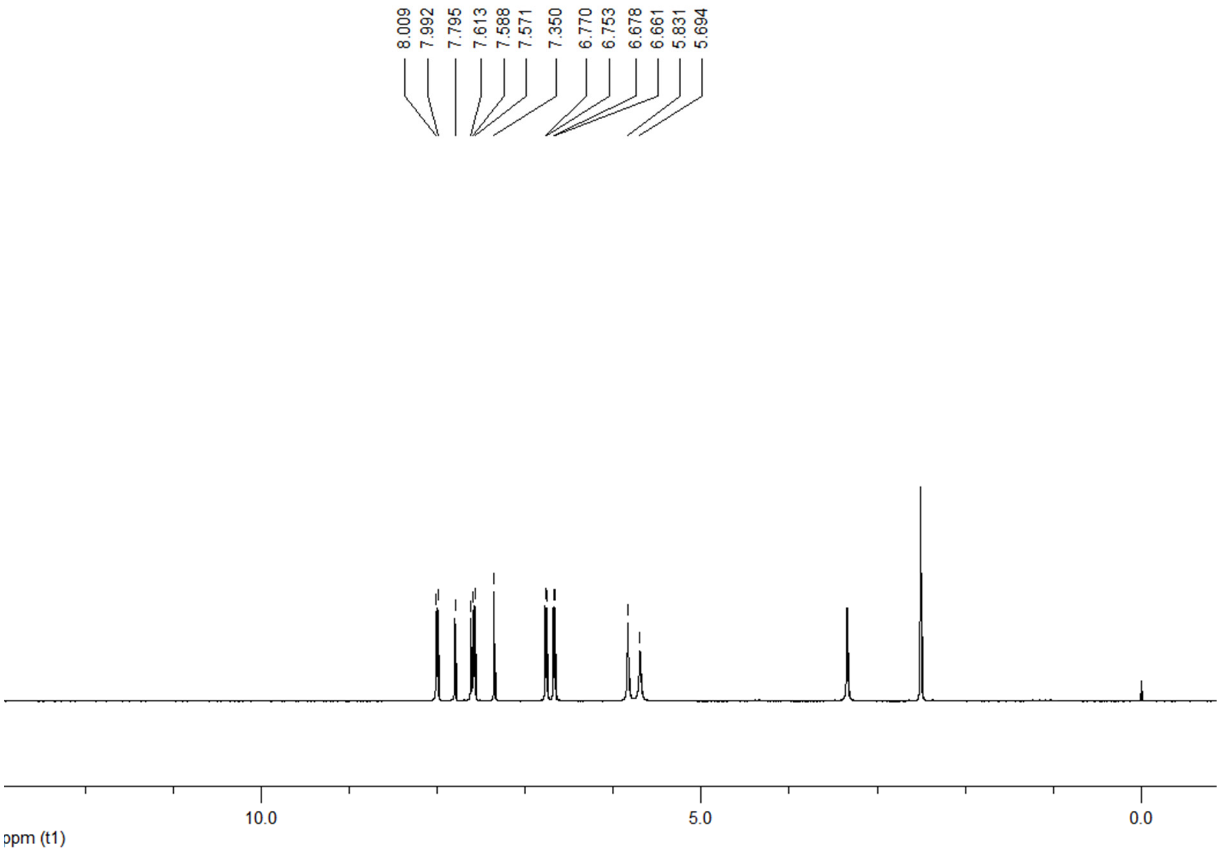

Figure S1-1. ^{1}H NMR spectrum of the **7a**.

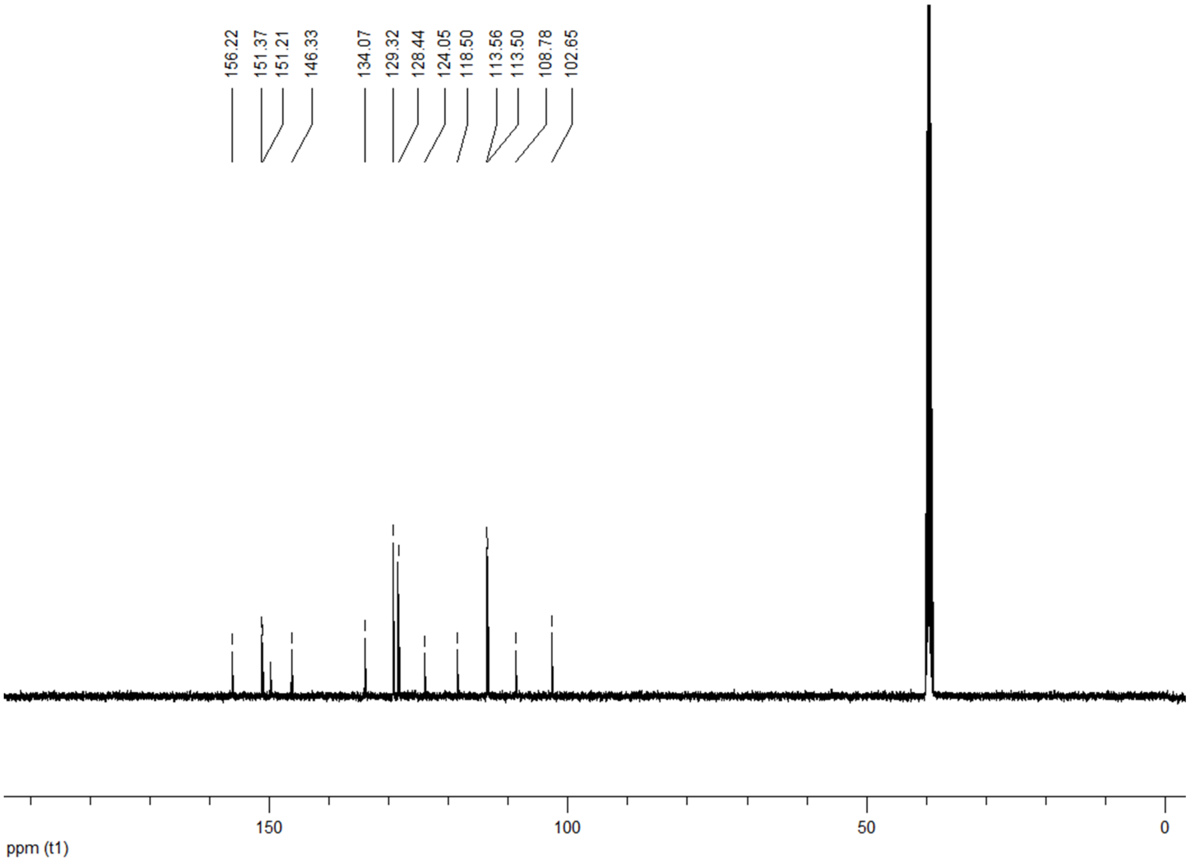

Figure S1-2. ^{13}C NMR spectrum of the **7a**.

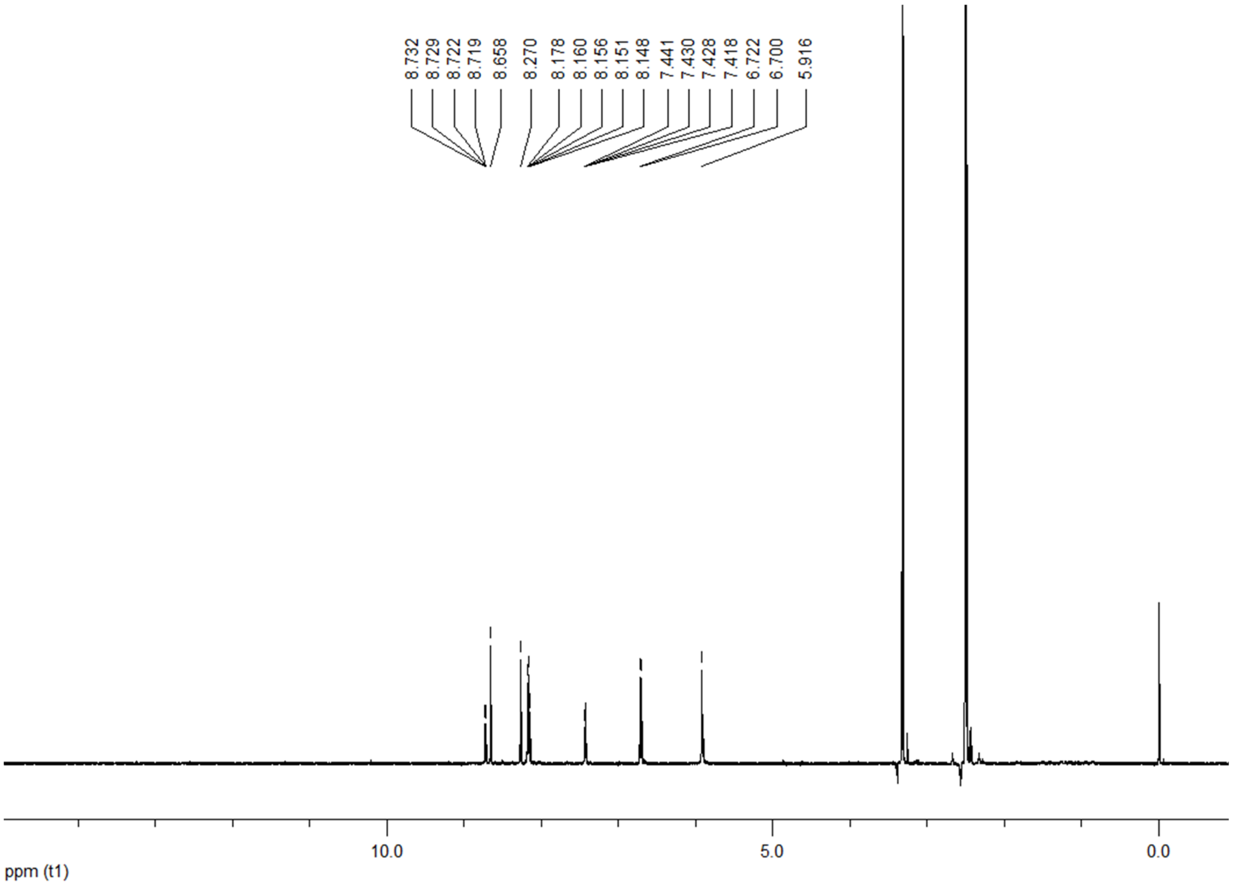

Figure S2-1. ^{1}H NMR spectrum of the **7b**.

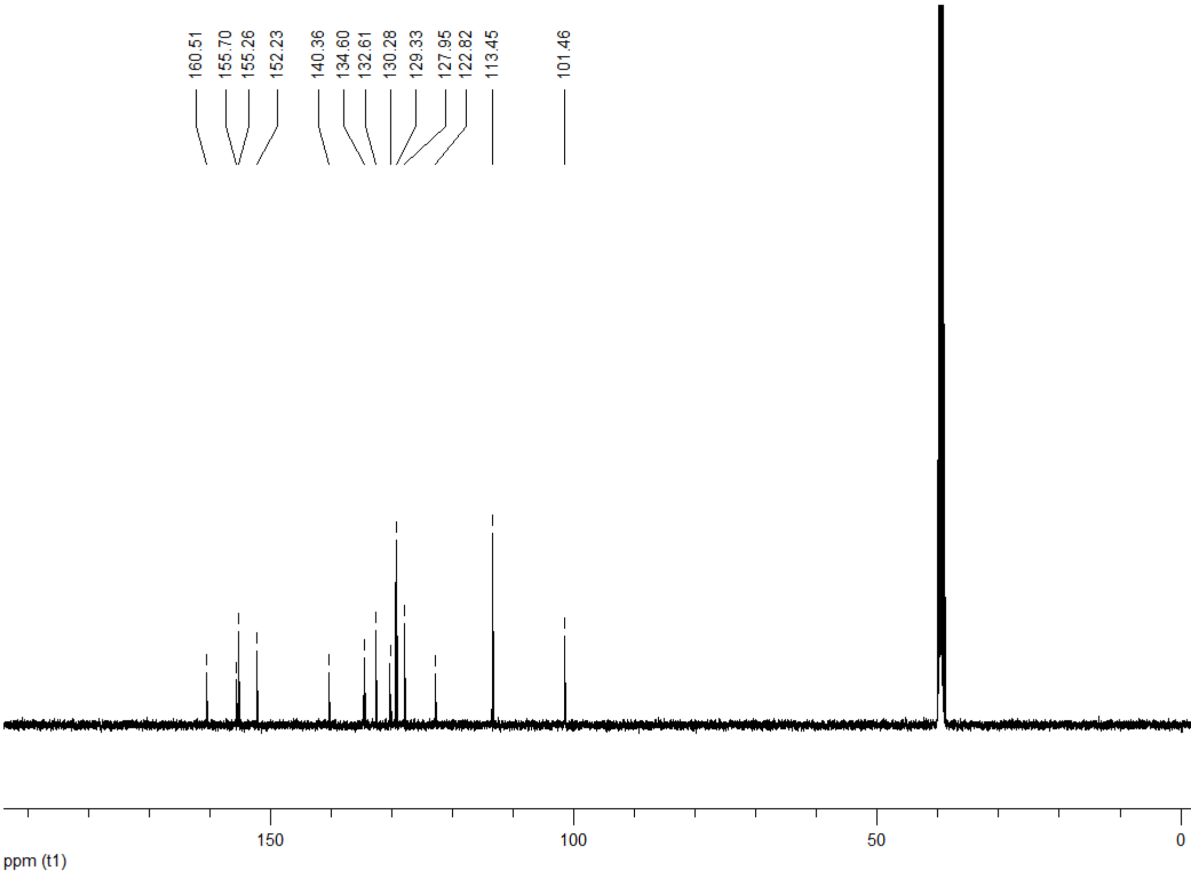

Figure S2-2. ^{13}C NMR spectrum of the **7b**.

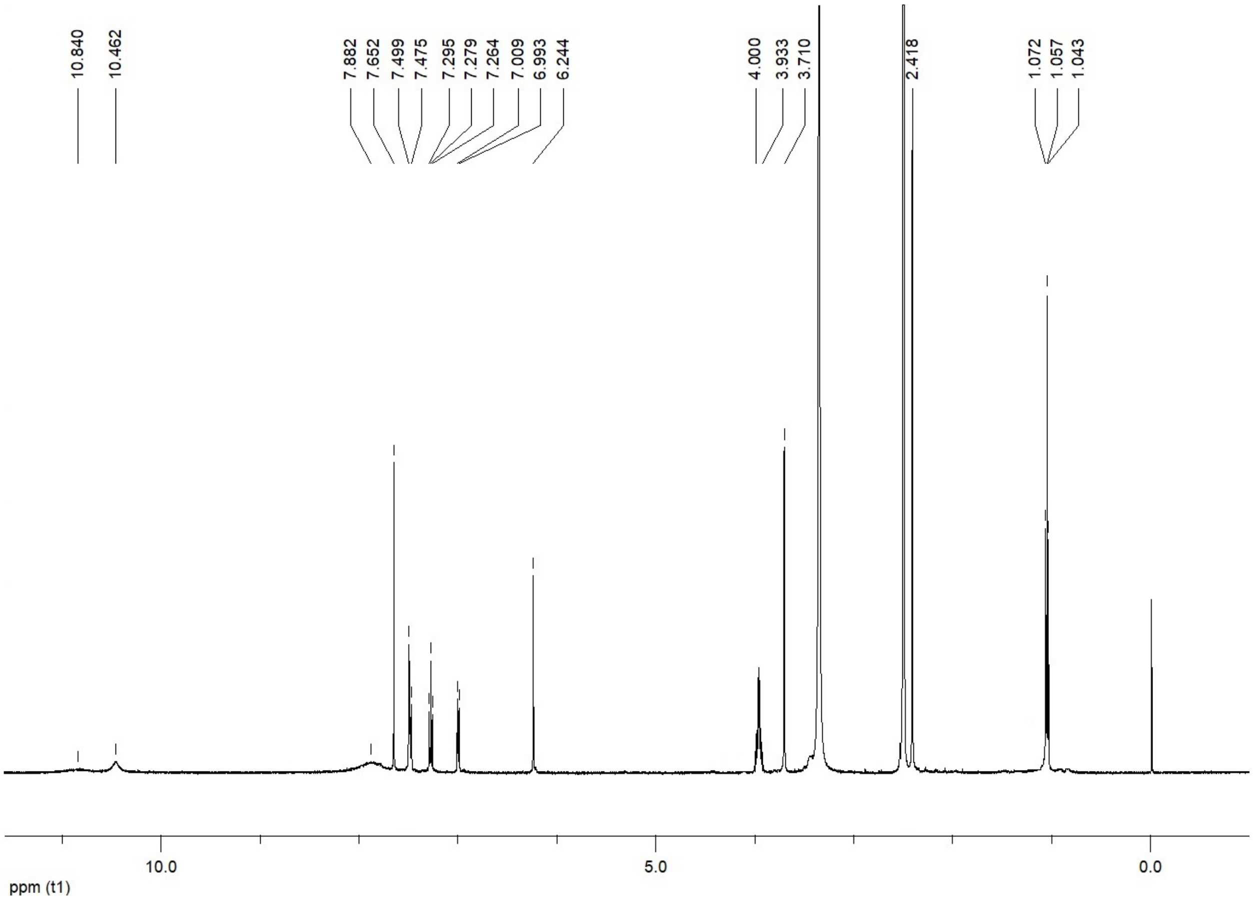

Figure S3-1. ^{1}H NMR spectrum of the **10a**.

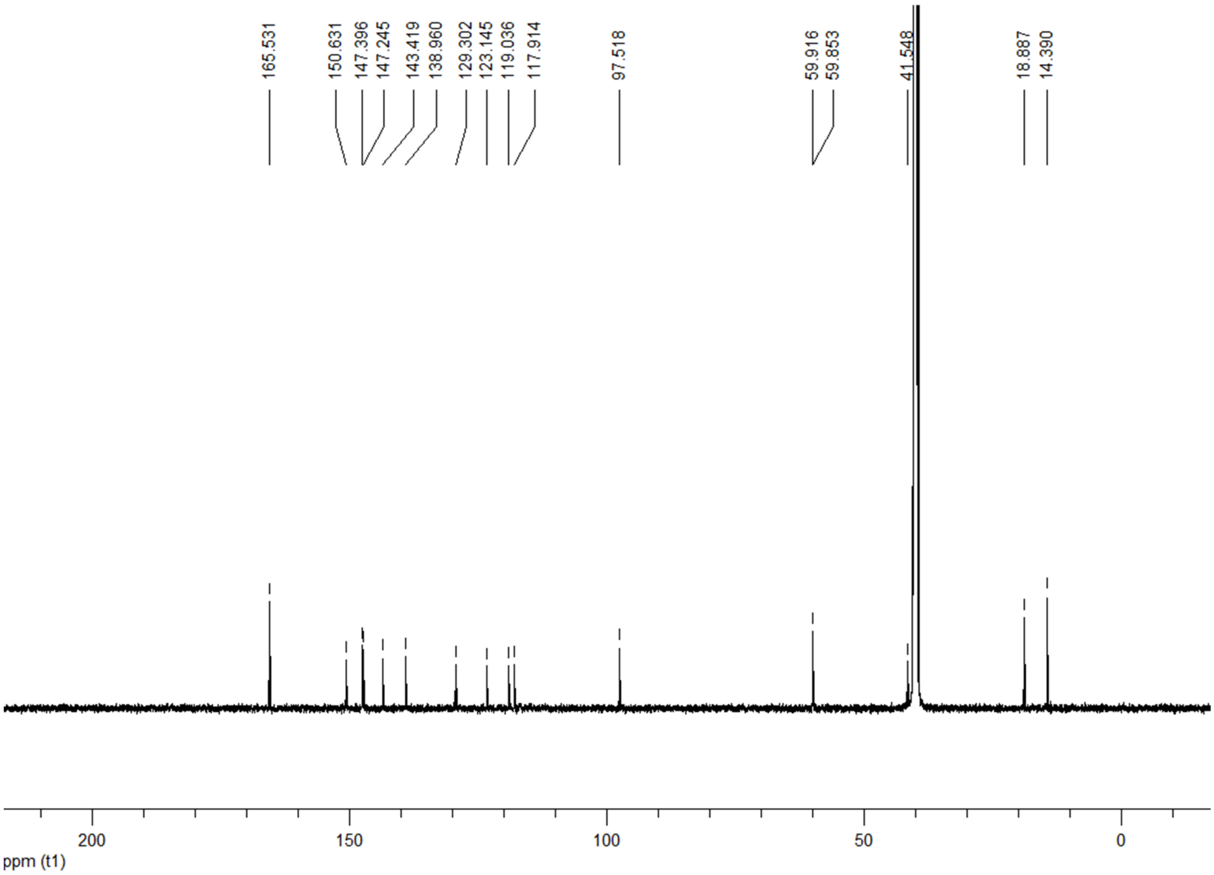

Figure S3-2. ^{13}C NMR spectrum of the **10a**.

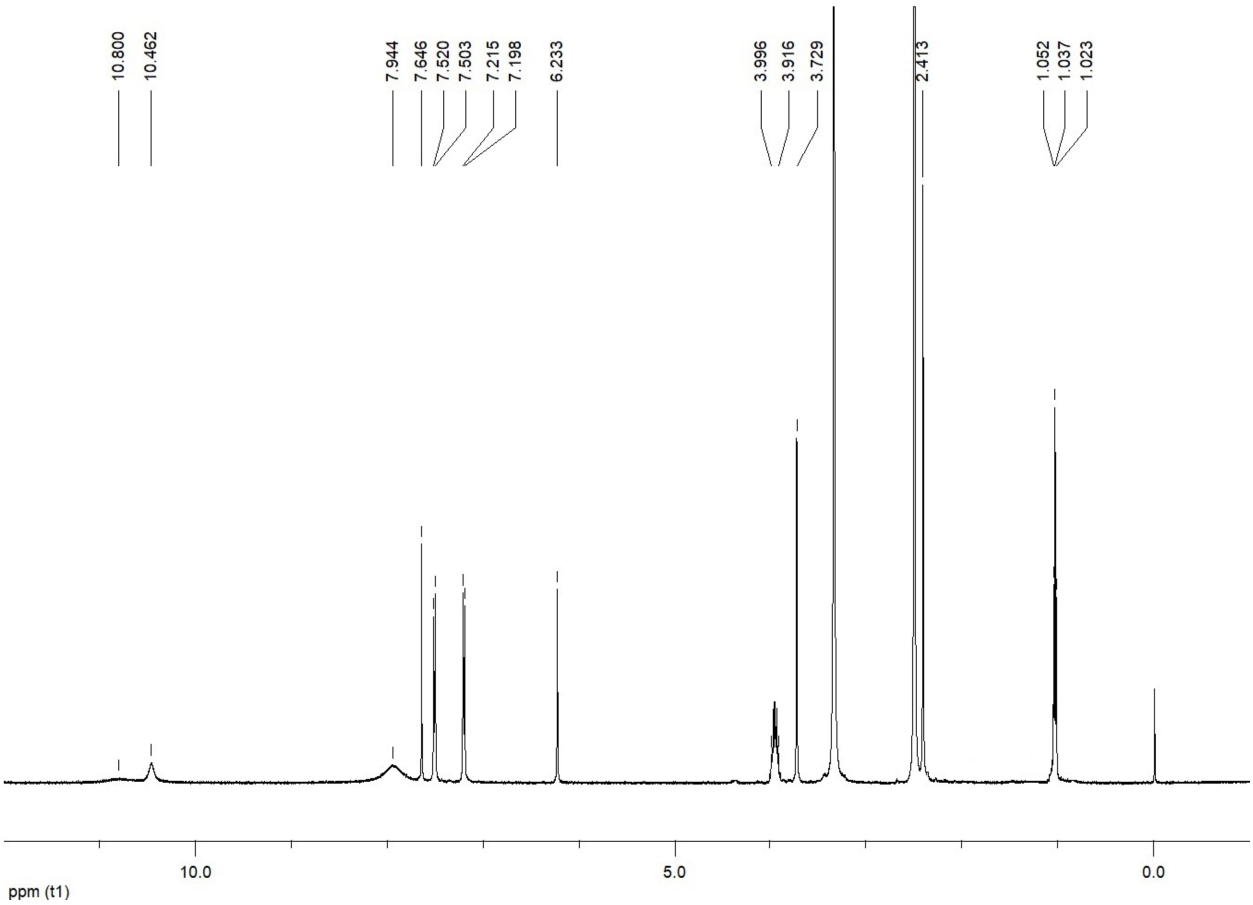

Figure S4-1. ^{1}H NMR spectrum of the **10b**.

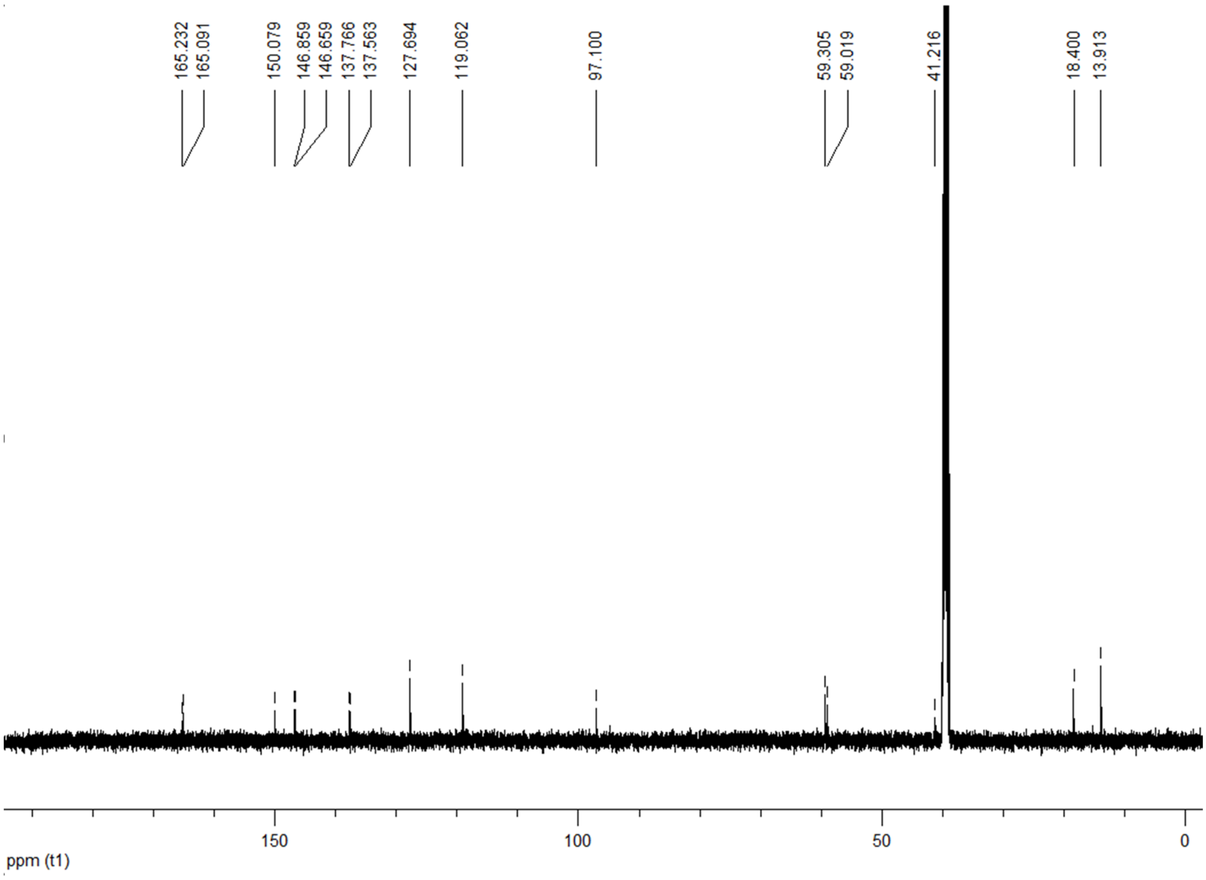

Figure S4-2. ^{13}C NMR spectrum of the **10b**.

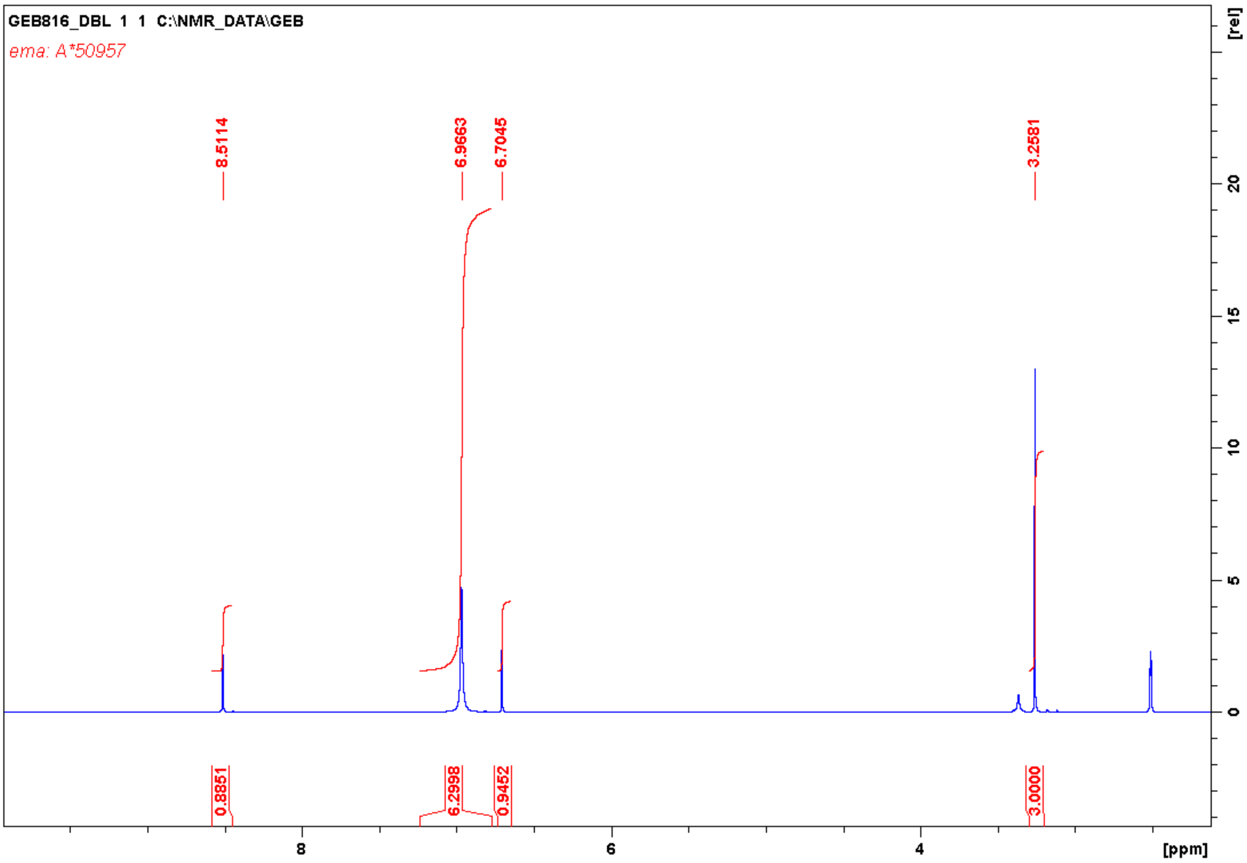

Figure S5-1. ^{1}H NMR spectrum of the **12a**.

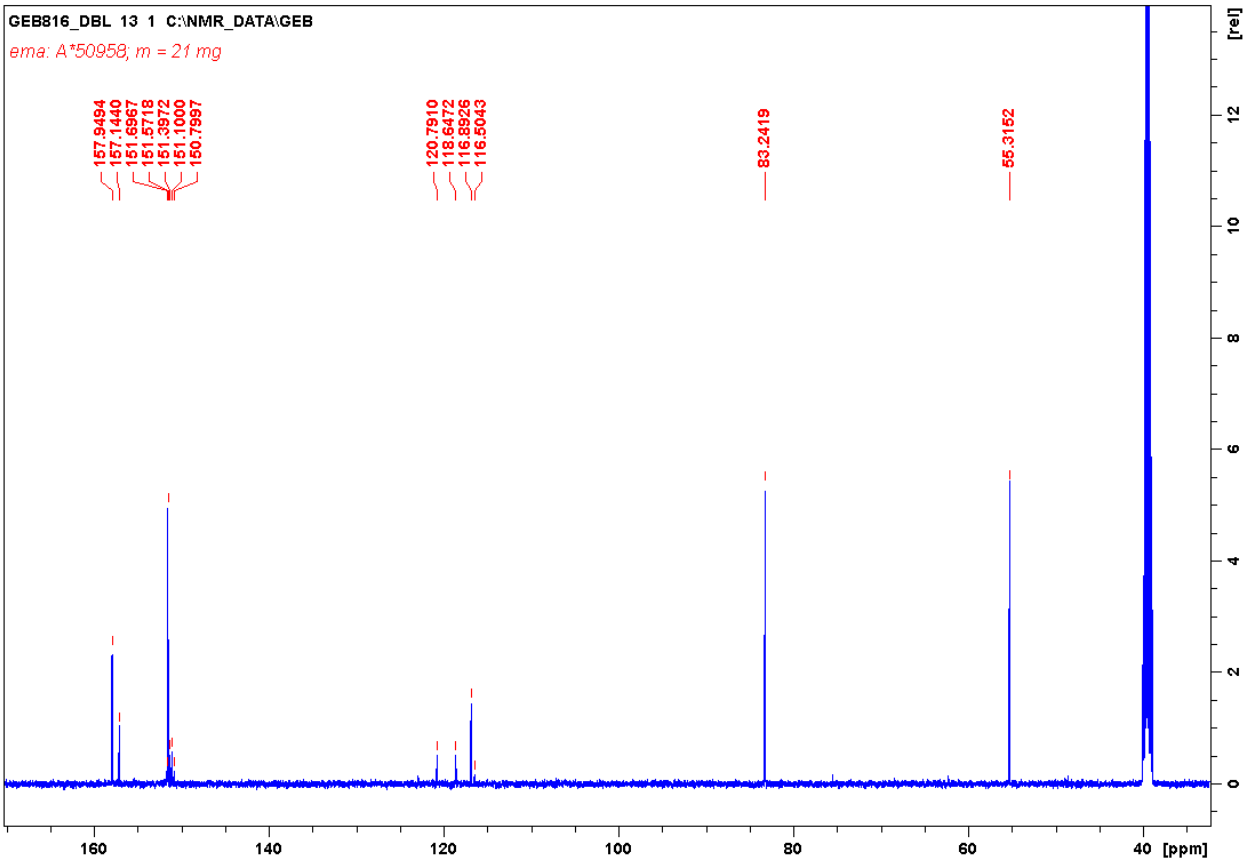

Figure S5-2. ^{13}C NMR spectrum of the **12a**.

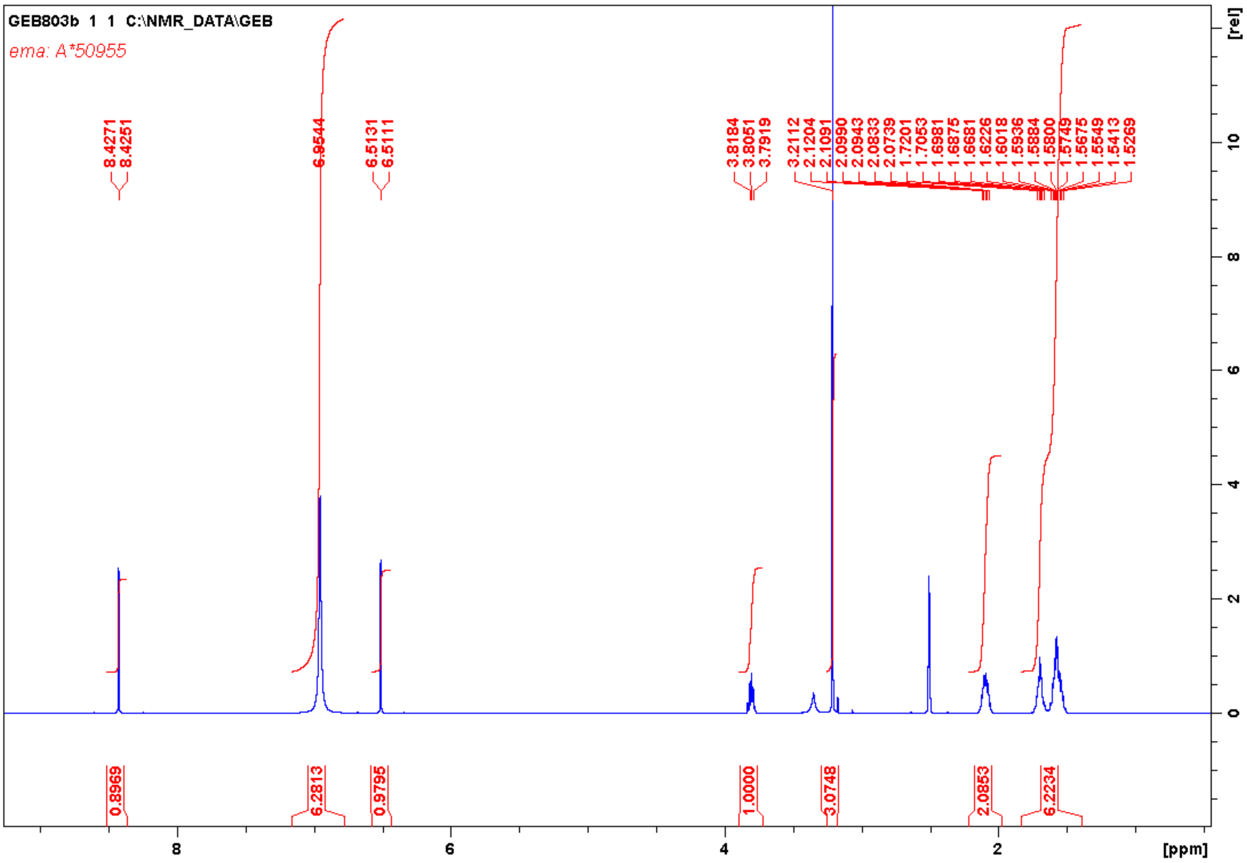

Figure S6-1. ^{1}H NMR spectrum of the **12b**.

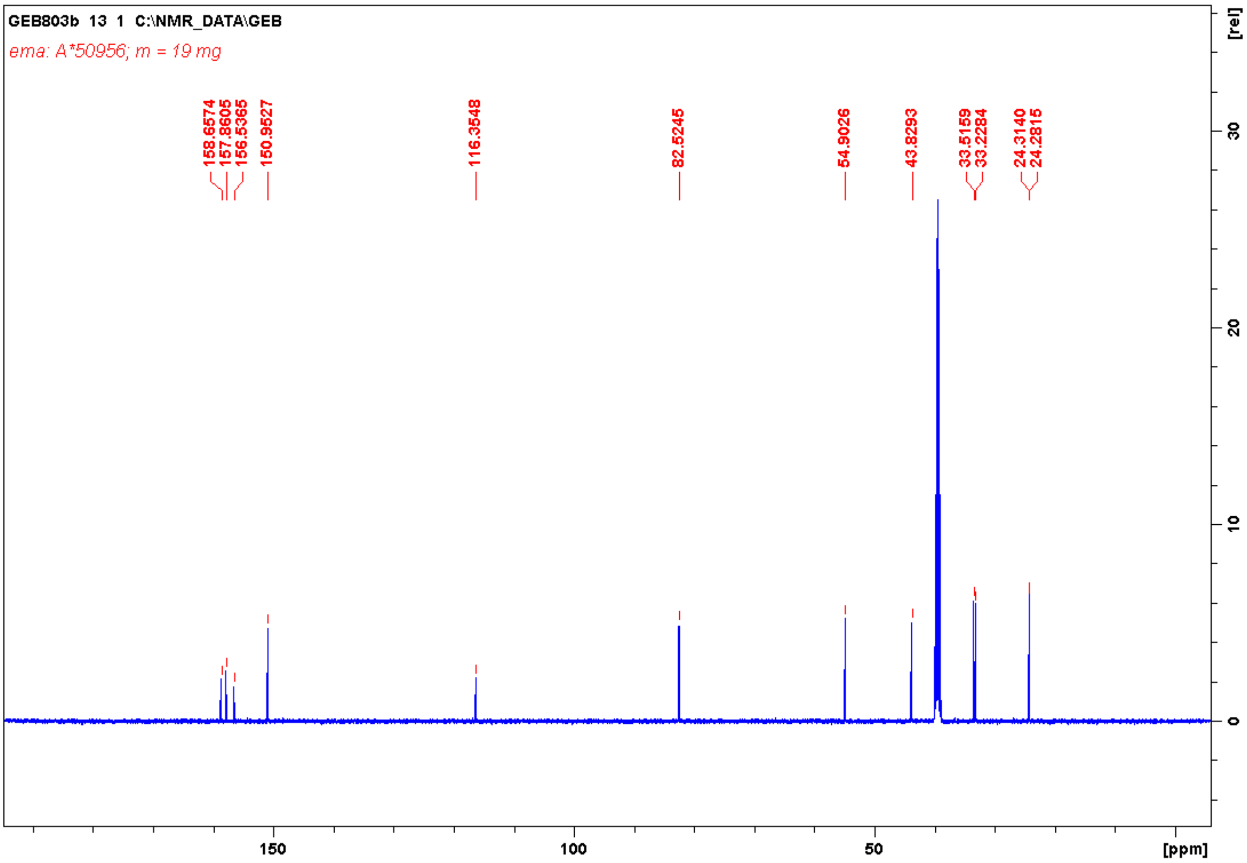

Figure S6-2. ^{13}C NMR spectrum of the **12b**.

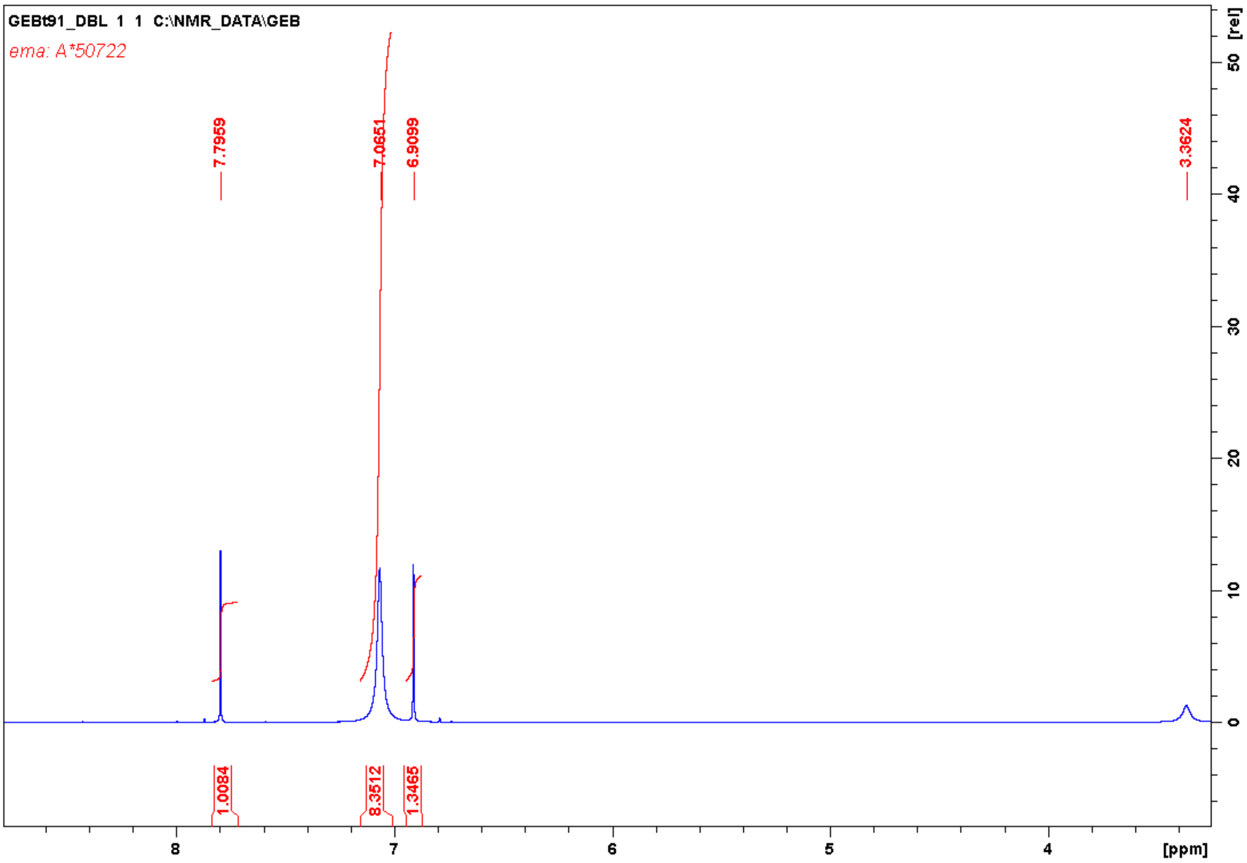

Figure S7-1. ^{1}H NMR spectrum of the **14**.

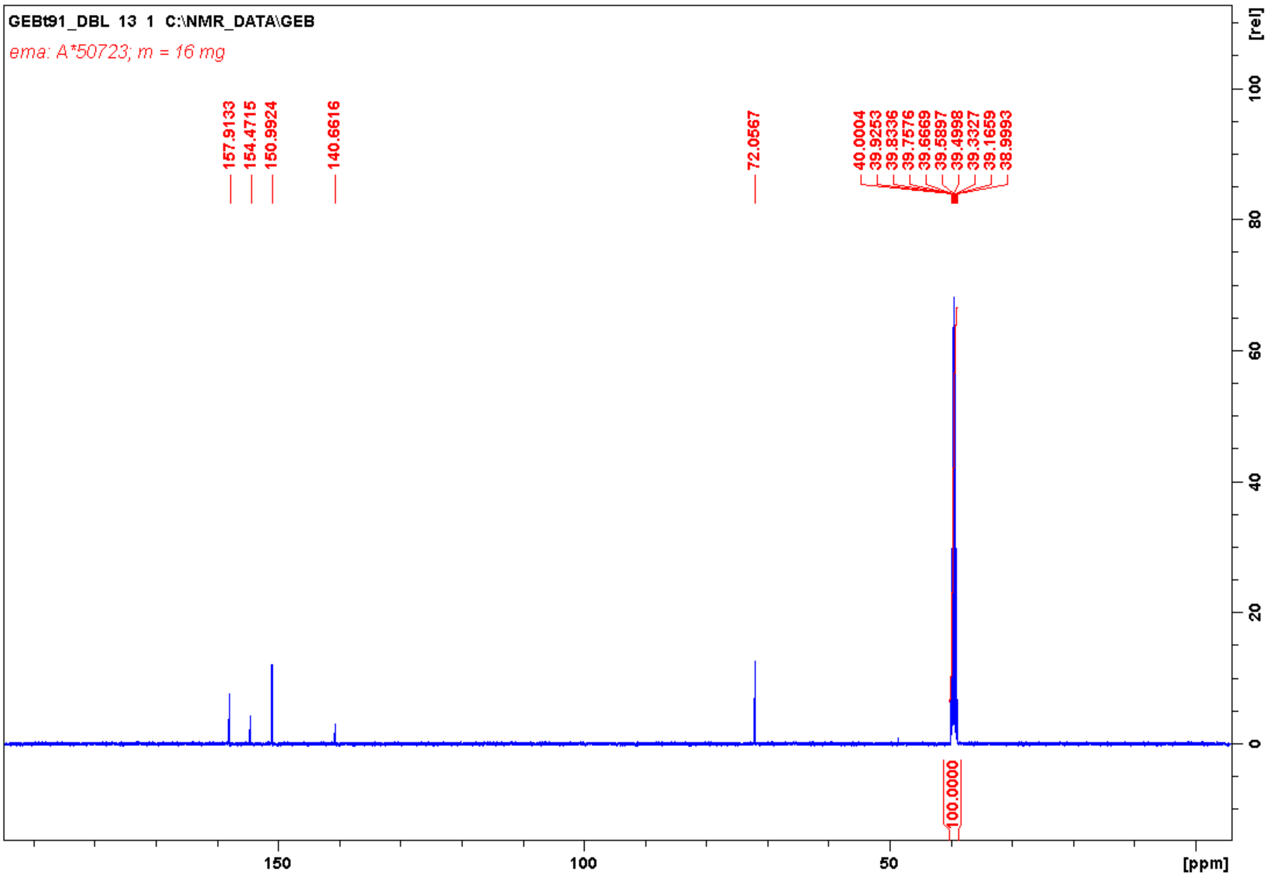

Figure S7-2. ^{13}C NMR spectrum of the **14**.

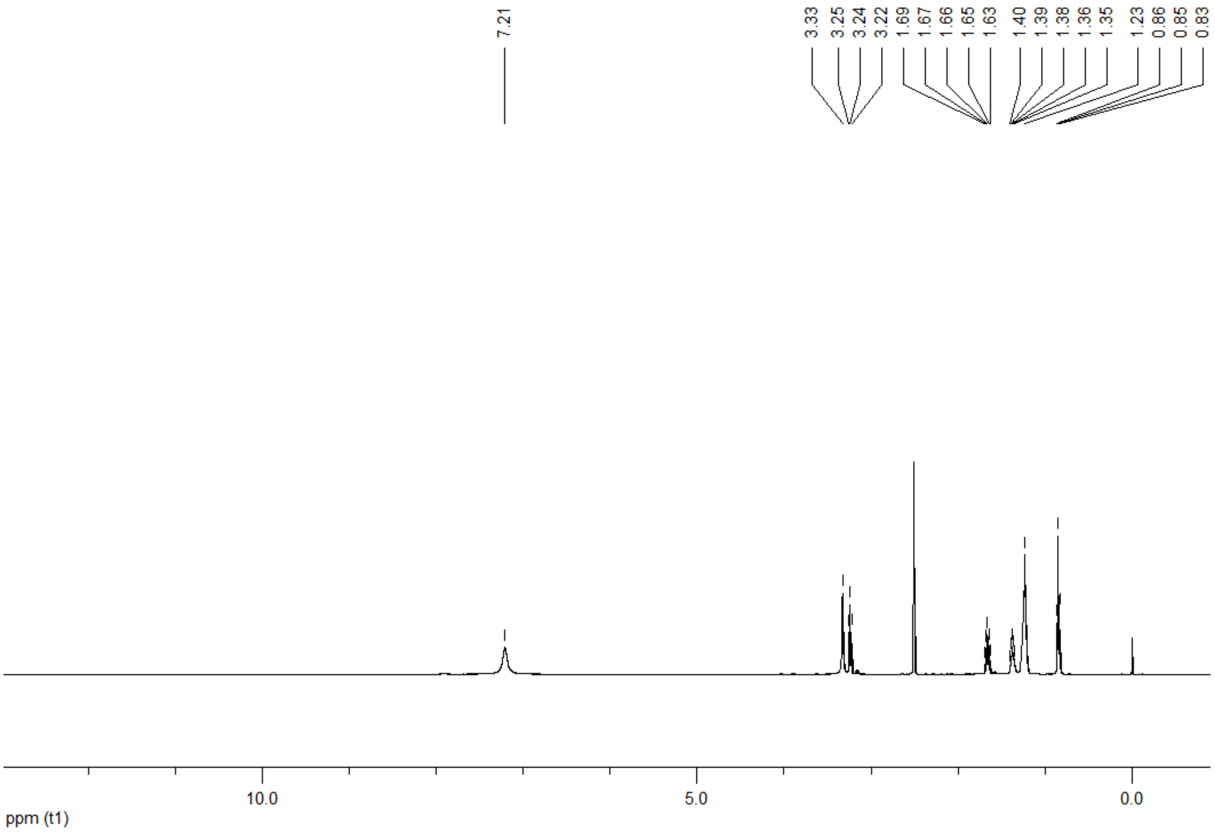

Figure S8-1. ^{1}H NMR spectrum of the **16a**.

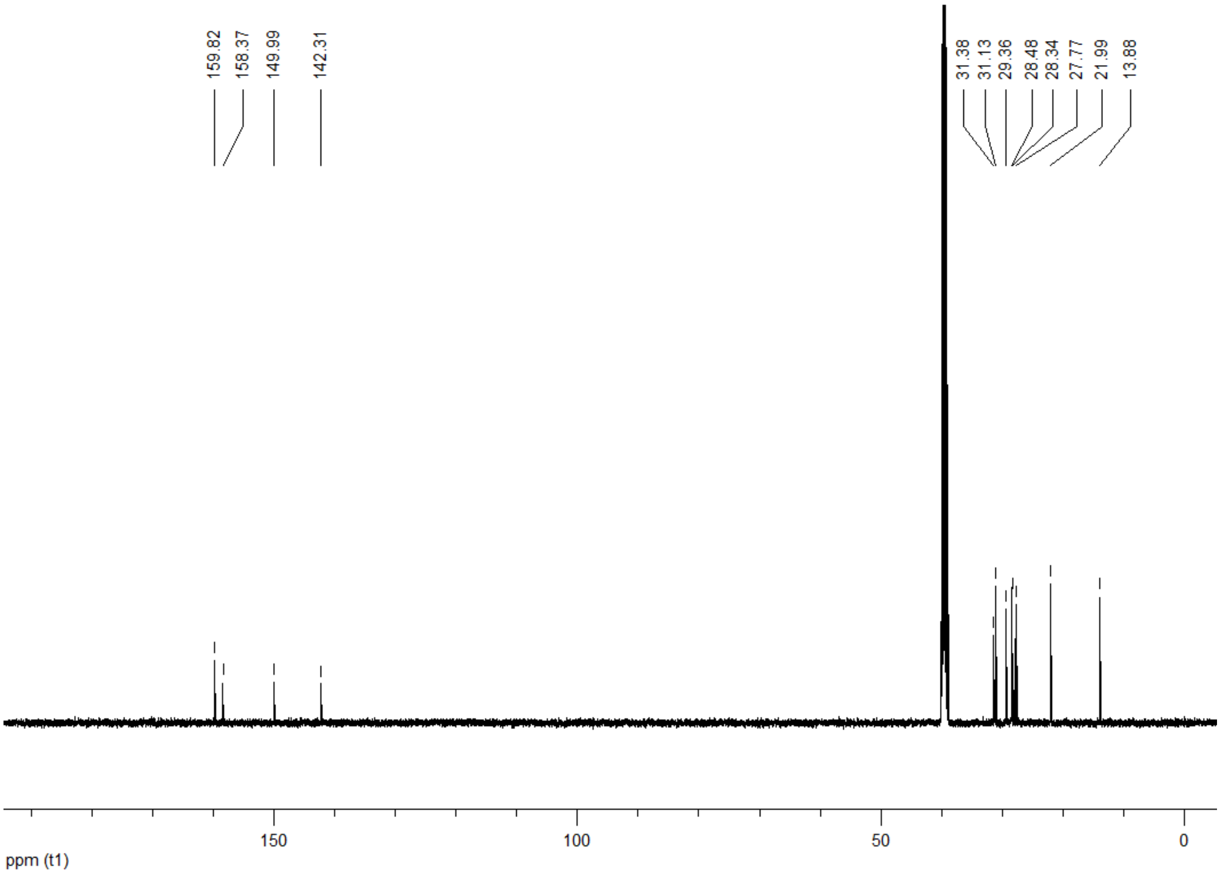

Figure S8-2. ^{13}C NMR spectrum of the **16a**.

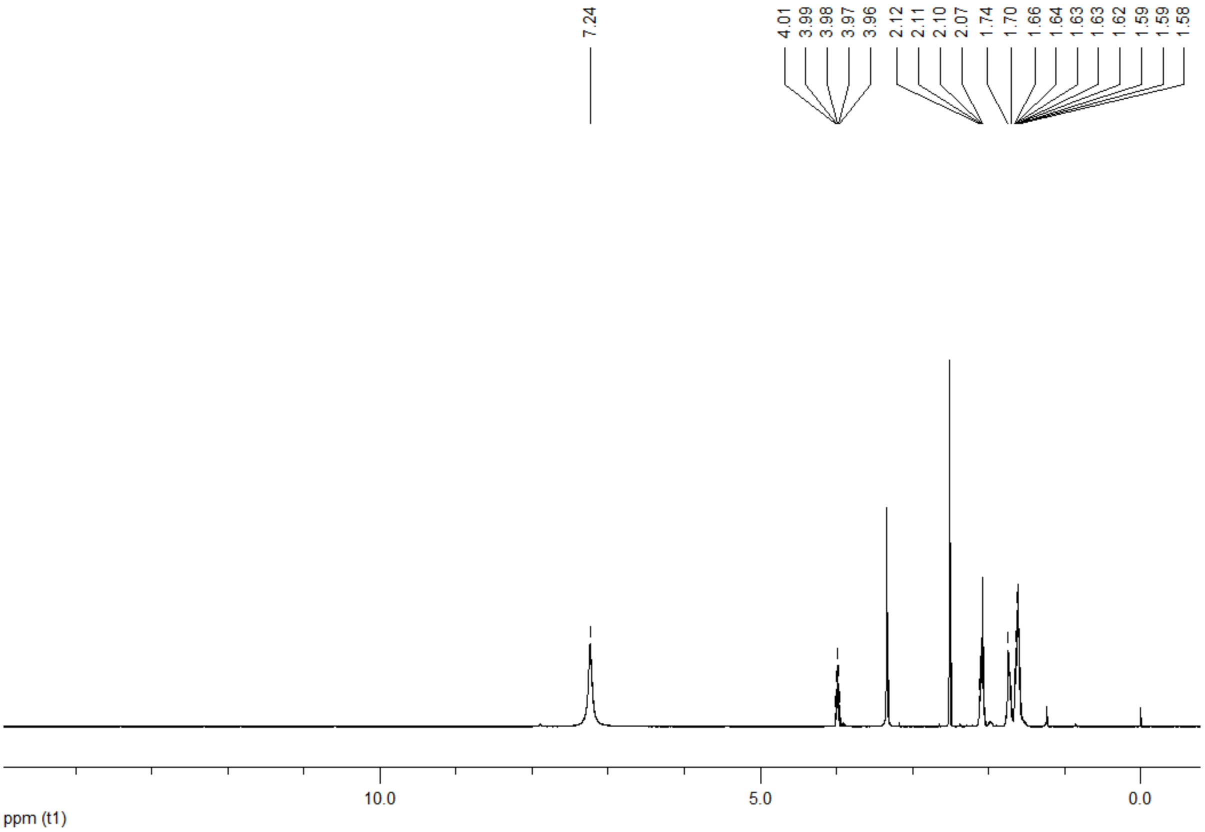

Figure S9-1. ^{1}H NMR spectrum of the **16b**.

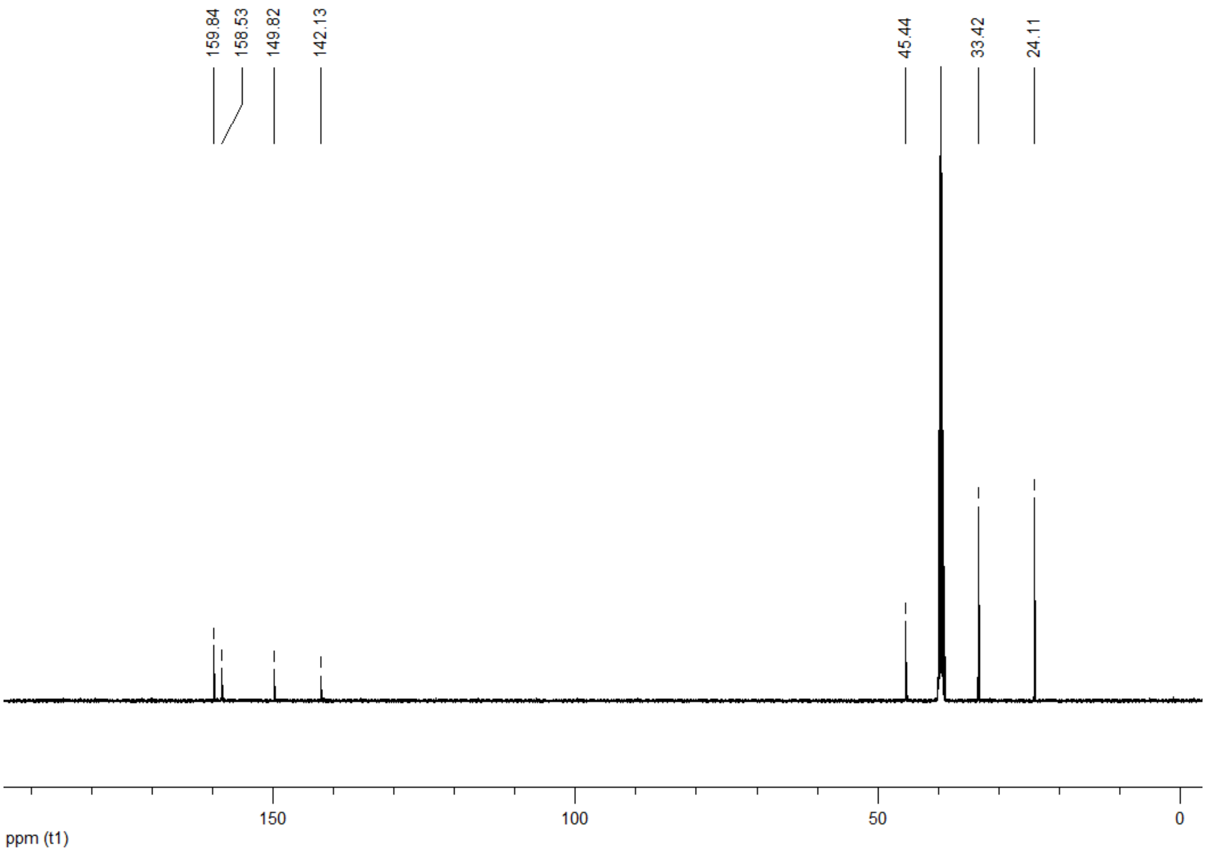

Figure S9-2. ^{13}C NMR spectrum of the **16b**.

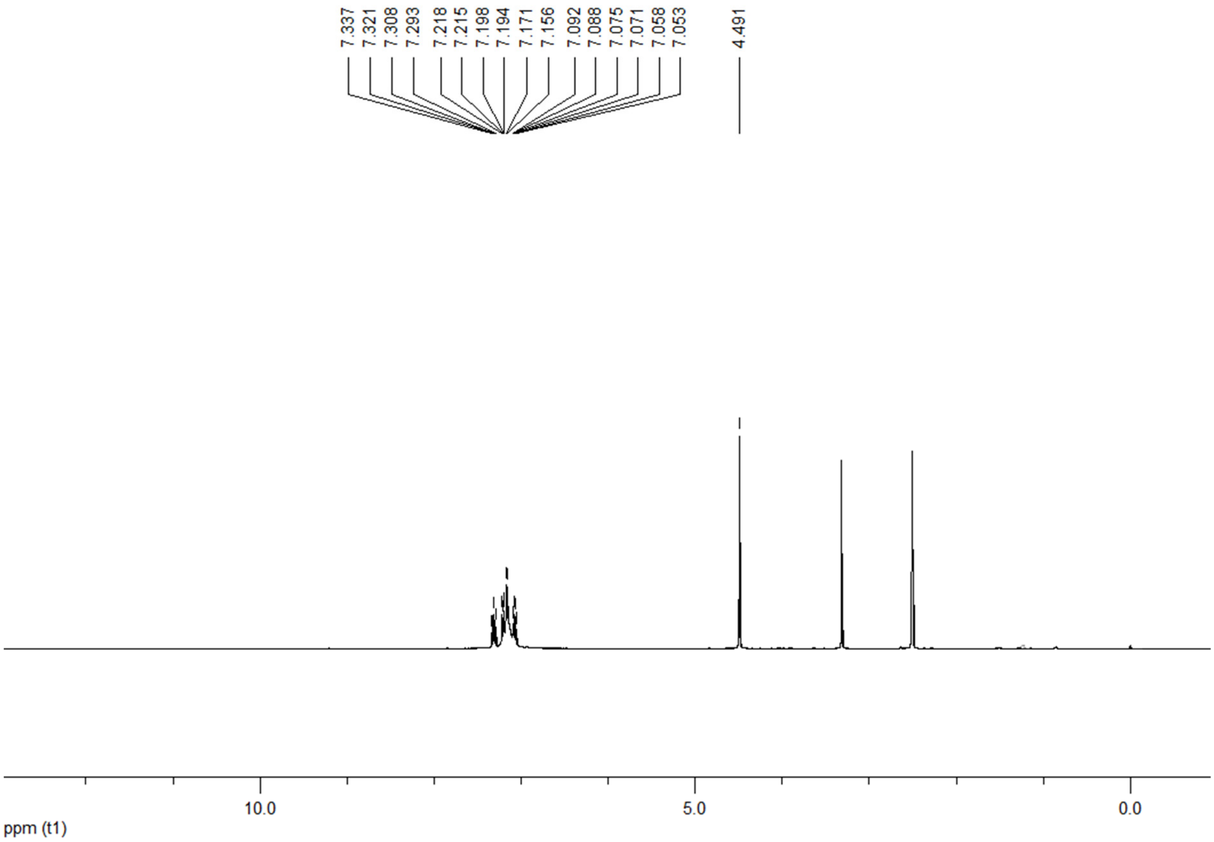

Figure S10-1. ^{1}H NMR spectrum of the **16c**.

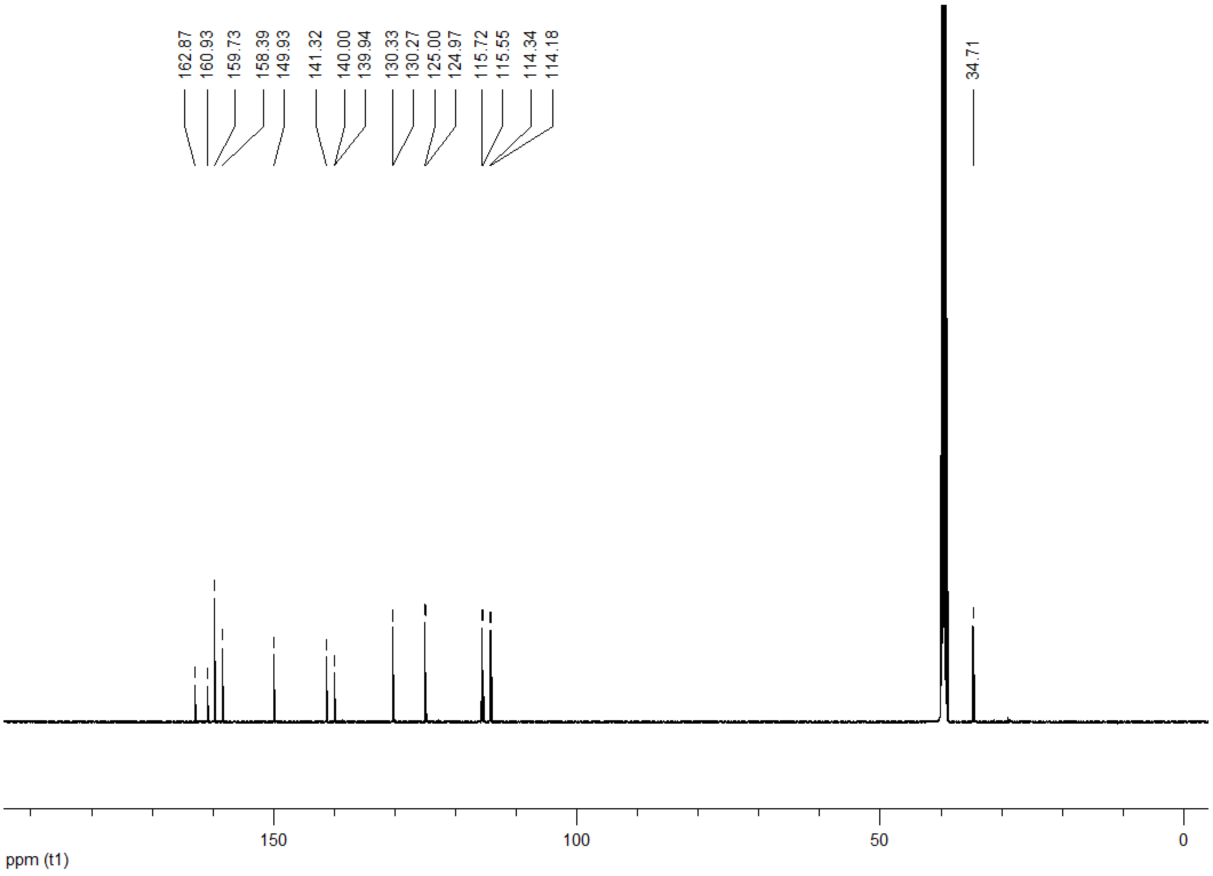

Figure S10-2. ^{13}C NMR spectrum of the **16c**.
